# Differential effectiveness of dietary zinc supplementation with autism-related behaviours in *Shank2* knockout mice

**DOI:** 10.1098/rstb.2023.0230

**Published:** 2024-06-10

**Authors:** Kevin Lee, Yewon Jung, Yukti Vyas, Zoe Mills, Laura McNamara, Johanna M. Montgomery

**Affiliations:** ^1^ Department of Physiology and Centre for Brain Research, University of Auckland, Auckland, New Zealand

**Keywords:** zinc, shank, synapse, autism

## Abstract

The family of SHANK proteins have been shown to be critical in regulating glutamatergic synaptic structure, function and plasticity. *SHANK* variants are also prevalent in autism spectrum disorders (ASDs), where glutamatergic synaptopathology has been shown to occur in multiple ASD mouse models. Our previous work has shown that dietary zinc in *Shank3^−/−^* and *Tbr1^+/−^* ASD mouse models can reverse or prevent ASD behavioural and synaptic deficits. Here, we have examined whether dietary zinc can influence behavioural and synaptic function in *Shank2^−/−^* mice. Our data show that dietary zinc supplementation can reverse hyperactivity and social preference behaviour in *Shank2^−/−^* mice, but it does not alter deficits in working memory. Consistent with this, at the synaptic level, deficits in NMDA/AMPA receptor-mediated transmission are also not rescued by dietary zinc. In contrast to other ASD models examined, we observed that SHANK3 protein was highly expressed at the synapses of *Shank2^−/−^* mice and that dietary zinc returned these to wild-type levels. Overall, our data show that dietary zinc has differential effectiveness in altering ASD behaviours and synaptic function across ASD mouse models even within the *Shank* family.

This article is part of a discussion meeting issue 'Long-term potentiation: 50 years on'.

## 1. Introduction

SHANK proteins play an integral structural and functional role in excitatory glutamatergic synapses, acting as master-scaffolding proteins within the post-synaptic density (PSD). Consequently, autism spectrum disorder (ASD)-associated mutations in *Shank* genes profoundly and detrimentally affect synaptic function and overall neurological development. Since 2010, mutations in the human *SHANK2* gene have been linked to neurodevelopmental disorders: in individuals with ASD, both inherited and de novo mutations are observed, manifesting as missense variations, duplications, microdeletions and premature truncations of the *SHANK2* gene [1–5]. *SHANK2* single nucleotide variants (SNVs) affecting highly conserved amino acid sites found in ASD patients cause a significant reduction in synapse density as well as miniature excitatory post-synaptic current (mEPSC) frequency when expressed in rodent-dissociated hippocampal neurons [3,6]. Furthermore, Shank2-deletion (*Shank2*^−/−^) mouse models of ASD have been reported to present deficits in social interactions, reduced ultrasonic vocalizations, increased anxiety and hyperactivity [7,8].

SHANK2 proteins have been shown to be zinc-sensitive [9], where the SHANK–zinc interaction enables the formation of large SHANK scaffolding sheets within the PSD, creating a platform for the structural and functional interactions of other proteins [10]. Zinc deficiency, therefore, affects several neurological functions and neuronal maturation, and prenatal zinc-deficient mice display characteristic ASD behaviours such as impaired social behaviour, language and communication deficits and restricted repetitive behaviours [11,12]. Several studies have elucidated that prenatal and postnatal dietary zinc supplementation can correct neuronal and behavioural ASD-associated deficits in the *Shank3^−/−^* and *Tbr1^+/^*^−^ mouse models of ASD [13–15]. Additionally, *in vitro* chronic zinc exposure prevented structural and functional ASD-associated synaptic deficits caused by ASD-associated *Shank2* SNVs [6]. Therefore, in this study, we aimed to examine the potential of dietary zinc supplementation in correcting behavioural and physiological alterations observed in *Shank2^−/−^* mice. Our data show that dietary zinc supplementation can alter a subset of ASD behaviours in *Shank2^−/−^* mice but has limited effects on synaptic structure and function in the hippocampus, suggesting dietary zinc has differential effects in *Shank2^−/−^* mice compared with other ASD models.

## 2. Methods

### (a) Animals

All animal experimentation methods were approved by the University of Auckland Animal Ethics Committee (approval number 001969) and were in adherence with ARRIVE guidelines. The *Shank2*^−/−^ mice were provided by Professor Eunjoon Kim (KAIST, South Korea) and maintained at the Vernon Jansen Unit animal facility at the University of Auckland, Auckland, New Zealand. Wild-type *Shank2*^+/+^ (WT) and homozygous *Shank2*^−/−^ mice were generated from *Shank2*^+/−^ male and female breeding pairs. All experimental animals were housed under a standard 12/12 h light–dark cycle and weaned at 3 weeks after birth (P21), after which animals were housed in groups of 2–4 per individually ventilated cage with mixed genotypes. At P21 animals were randomly assigned to the control zinc diet (30 ppm (parts per million) zinc; D19410B; Research Diets, Brunswick, NJ, USA) or the zinc supplementation diet (150 ppm zinc; D06041101; Research Diets, Brunswick, NJ, USA) for 6–8 weeks, as described in our previous work [13,14,16]. Food and water were available ad libitum. Behavioural, electrophysiological and imaging experiments were performed at 9–11 weeks of age on male and female mice on either zinc diet. All experiments and analyses were conducted blind to genotype and zinc diet via a unique identification number allocated at weaning.

### (b) Behavioural testing

All behavioural tests were performed in an isolated room under the light cycle, using a ceiling camera DFK21AF04 (SDR Scientific) to record and monitor animal behaviour. ezTrack (open source) was used for generating heat maps and tracking animal movement. The grooming test arena and three-chamber apparatus were washed with 70% ethanol and then 3% acetic acid between each behavioural trial. The same cohort of animals underwent all tests over a period of 1–2 weeks. No two behavioural tests were performed on the same day.

To assess movement and hyperactivity, each mouse was placed in a cylindrical arena (17 cm radius) and habituated for 10 min under red light conditions (15 lux). The amount and speed of movement were recorded and analysed over 30 min. To assess social interaction, the three-chamber apparatus was used under low light conditions (15 lux) as described in our previous work [13,14,16]. Briefly, a mesh container was placed in both the left and right chambers, and then each mouse was placed in the middle chamber. The doors between the chambers were then opened to assess mouse exploration in all three chambers. After 10 min, the mouse was guided back to the centre chamber. In phase 2, a sex- and age-matched stranger mouse (stranger 1: S1) was placed in one of the containers, while the other chamber’s container remained empty (empty cup: E). The test mouse was then allowed to explore freely for 10 min. The social interaction preference index was calculated by the time spent in close interaction with S1 subtracted by time spent in close interaction with the empty cup in the chamber. The Y-maze test was used to assess working memory. Mice were placed in the centre of the Y-maze (arm length 50 cm, width 10 cm and height 15 cm) and given 5 min to freely explore two arms of the maze. The third arm was then made available for exploration, and the time spent in the novel arm was recorded as well as the number of transitions to the novel arm.

### (c) Electrophysiology

Whole-cell patch-clamp recordings were performed as described in our previous work in ASD mouse models [13,14,16]. Briefly, acute coronal brain slices were prepared from 9- to 11-week-old mice after euthanasia via CO_2_ and decapitation. The brain was transferred to ice-cold carbogenated (95% O_2_, 5% CO_2_) protective cutting artificial cerebrospinal fluid (aCSF): (in mM), 93 *N*-methyl-d-glucamine (NMDG), 2.5 KCl, 1.25 NaH_2_PO_4_, 30 NaHCO_3_, 20 HEPES, 25 glucose, 2 thiourea, 5 L-ascorbic acid, 3 Na pyruvate, 0.5 CaCl_2_, 10 MgSO_4_.7H_2_O, pH 7.4 and 295–305 mOsm. Brain slices were generated at 300 µm thickness using a vibratome (VT1200S, Leica Biosystems; 1 mm *x*-plane blade vibration, 0.05 mm min^–1^ *y*-plane advancement speed, 0 mm z-axis vibration calibration), and then slices were transferred to protective cutting aCSF at 34°C. Prior to recording, slices were maintained at room temperature (RT, 24°C) for a minimum of 1 h, submerged in a holding chamber containing carbogenated recovery aCSF: (in mM) 97 NaCl, 2.5 KCl, 1.2 NaH_2_PO_4_, 30 NaHCO_3_, 25 glucose, 20 HEPES, 2 CaCl_2_, 2 MgSO_4_.7H_2_O, 2 thiourea, 5 L-ascorbic acid, 3 Na pyruvate, pH 7.4 and 295–305 mOsm.

Slices were transferred to the recording chamber superfused with carbogenated recording aCSF: (in mM) 119 NaCl, 2.5 KCl, 1 Na_2_HPO_4_, 1.3 MgSO_4_, 26.2 NaHCO_3_, 11 D-(+)-glucose, 2.5 CaCl_2_, pH 7.4 and 305–310 mOsm at RT at 2–3 ml min^–1^. The pyramidal neurons of the CA1 region were visualized using infrared differential interference contrast (IR-DIC) optics with a 40 × water immersion objective lens mounted on an Olympus BX51 microscope (Olympus Corporation, Japan). A platinum–iridium concentric bipolar stimulating electrode was placed in stratum radiatum and stimulation was performed with a constant isolated current stimulator (Model DS3; Digitimer, USA) with a duration of 500 μs at 0.05 Hz frequency. Whole-cell patch-clamp recordings of CA1 pyramidal neurons were acquired with glass recording electrodes (borosilicate tubing filamented glass electrodes, BF150–86-7.5; Sutter Instrument Company, USA) of 5–7 MΩ resistance pulled by a vertical electrode puller (PC-10; Narishige, Japan) and filled with an internal solution: (in mM) 120 Cs gluconate, 40 HEPES, 5 MgCl_2_, 2 NaATP, 0.3 NaGTP, 5 QX314, pH 7.2 and 298 mOsm. Electrophysiological signals were amplified (MultiClamp 700B; Axon Instruments, USA) and digitized (Digidata 1550B; Axon Instruments, USA). Events were sampled at 20 kHz and low-pass filtered at 2 kHz. All data were obtained and analysed using pCLAMP 10 acquisition software and Clampfit 10, respectively (Axon Instruments, USA). Series resistance (Rs) was monitored before and after each recording paradigm, and any recordings with Rs variation over 20% or over 25 MΩ were discarded from the analysis.

To measure the NMDA/AMPA ratio, CA1 pyramidal neurons were first voltage-clamped at –70 mV, and 30 consecutive AMPAR-mediated EPSCs at a stimulus intensity that stably evoked 200–350 pA current responses were recorded (0.05 Hz) to ensure consistent pre-synaptic stimulation and AMPAR-mediated responses when measuring NMDAR EPSCs. Then, 10 μM 6-cyano-7-nitroquinoxaline-2,3-dione (CNQX6) was bath applied to block AMPAR-mediated current responses, and the holding potential was switched to +40 mV to record NMDAR-mediated EPSCs (30 sweeps, 0.05 Hz). The NMDA/AMPA ratio was calculated as the average peak amplitude of NMDAR-mediated EPSCs divided by the average peak amplitude of AMPAR-mediated EPSCs. Picrotoxin (100 µM) was included in the recording aCSF to block GABA_A_ receptor-mediated inhibitory currents during stimulation. Isolated AMPAR-mediated EPSCs were measured at the half-maximal EPSC amplitude.

### (d) Immunohistochemistry

Immunostaining for SHANK3 and synapsin1/2 was performed as previously described [13,14]. Coronal sections (50 µm) were permeabilized (0.25% Triton X-100 in 1 × phosphate-buffered saline (PBST), pH 7.4) at 4°C and non-specific binding blocked with 10% normal goat serum (NGS) in 1 × PBST for 1 h at RT. The sections were immunolabelled for SHANK3 (1:500; 162 302, Synaptic Systems) and synapsin1/2 (1:500; 106 004, Synaptic Systems) in 1 × PBST containing 1% NGS for 72 h at 4°C then incubated for 4 h at RT with secondary antibodies (IgG‐Alexa Fluor 594, 1:500 or IgG‐Alexa Fluor 647, 1:500). Sections were mounted on microscope slides (Menzel Glaser) in Citifluor mounting medium (Agar Scientific, AF1). Hippocampal CA1 immunostaining was imaged via confocal microscopy (FV1000; Olympus Corporation, Japan) at 63× magnification (UPLSAPO, 1.35 NA) with 3× digital zoom using FluoView 4.0 image acquisition software, yielding images with a size of 512 × 512 pixels (0.138 µm pixel^–1^). Z-stacks were obtained (0.3 µm apart), and puncta density and colocalization analysis were performed using the ImageJ software (NIH, USA). Background intensity variation was corrected by subtracting a Gaussian (σ = 3 pixels) blurred version of the substack. The number, intensity and colocalization of SHANK3 and synapsin1/2 puncta were analysed by the 3D Objects Counter tool. SHANK3 puncta that colocalized with synapsin1/2 were defined as synaptic. Image analysis criteria were kept consistent for each experimental set, regardless of the genotype and diet manipulation. The puncta density (number of puncta per 1000 µm^3^) was compared between groups by normalizing to the average puncta density values calculated from the WT 30 ppm dietary zinc group.

### (e) Statistical analysis

All data in the text and figures are presented as mean ± standard error of the mean (s.e.m.). Statistical analyses were conducted using GraphPad Prism 9.0 and a *p* value of <0.05 was considered significant. Normality and homogeneity of data variance were assessed by the D'Agostino and Pearson normality test. Statistical significance was determined by two-way analysis of variance (ANOVA) with Šídák’s *post hoc* test, one-way ANOVA with Tukey’s *post hoc* multiple comparisons or non-parametric Kruskal–Wallis test, depending on data normality and the datasets compared. Significant results are marked with *p < 0.05, **p < 0.01, ***p < 0.001, ****p < 0.0001.

## 3. Results

Here, we sought to examine the effects of dietary zinc on ASD-related behaviours and synaptic function in *Shank2^−/−^* mice and determine whether any observed effects are similar or different from other ASD models previously tested.

**Figure 1.**
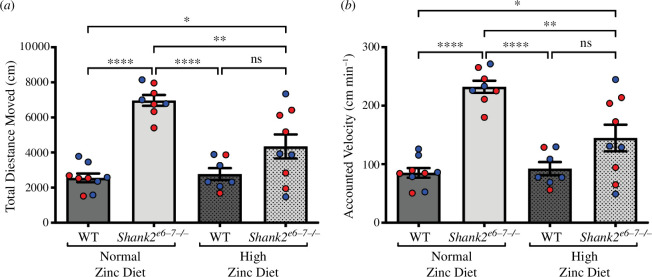
Hyperactivity is reversed by high dietary zinc in *Shank2^−/−^* mice. (*a*) Bar graph of total distance moved in WT (dark grey) and *Shank2^−/−^* (light grey) mice. *Shank2^−/−^* mice moved significantly further than WT mice. High dietary zinc reversed this phenotype, with *Shank2^−/−^* mice movement not significantly higher than WT mice fed high dietary zinc but now significantly lower than *Shank2^−/−^* mice fed the normal zinc diet. (*b*) Bar graph of the velocity of movement in WT (dark grey) and *Shank2^−/−^* (light grey) mice. *Shank2^−/−^* mice moved significantly faster than WT mice when on a normal zinc diet; however, high dietary zinc reversed this phenotype, with *Shank2^−/−^* mice velocity no longer significantly higher than WT mice fed high dietary zinc but now significantly lower than *Shank2^−/−^* mice fed the normal zinc diet. Each dot represents an individual animal. WT 30 ppm mice: *n* = 9; *Shank2^−/−^* 30 ppm mice: *n* = 8; WT 150 ppm mice: *n* = 7; and *Shank2^−/−^* 150 ppm mice: *n* = 9. Blue data points are male mice and red data points are female mice. All data were analysed using one-way ANOVA with Tukey’s *post hoc* test. ns = not significant. *p < 0.05, **p < 0.01, ****p < 0.0001.

*Shank2^−/−^* mice showed a hyperactivity phenotype as observed by an increase in distance moved and velocity of movement (figure 1*a* and *b*). Specifically, *Shank2^−/−^* mice moved on average 6968 ± 312.2 cm compared with WT controls 2560 ± 247.1 cm (*p* < 0.0001). The average velocity was 85.32 ± 8.236 cm min^–1^ for WT and 232.3 ± 10.41 cm min^–1^ for *Shank2^−/−^* mice (*p* < 0.0001). This hyperactivity phenotype was significantly decreased in *Shank2^−/−^* mice that were fed the supplemented zinc diet, where distance moved and average velocity were 2275 ± 336.5 cm and 95.52 ± 11.22 cm min^–1^ for WT 150 ppm mice and 4345 ± 684 cm and 144.8 cm min^–1^ for *Shank2^−/−^* mice fed 150 ppm (*p* = 0.0972 for total distance and velocity). Distance and velocity for *Shank2^−/−^* mice fed 150 ppm were significantly different from *Shank2^−/−^* mice fed the normal zinc diet (*p* < 0.001; figure 1*a* and *b*).

**Figure 2.**
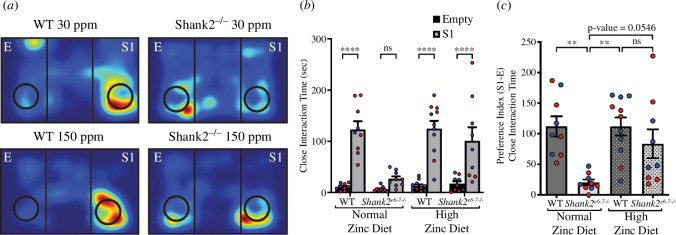
Social interaction and social preference ASD phenotypes are reversed by high dietary zinc in *Shank2^−/−^* mice. (*a*) Heat maps representing example movements of animals during the social interaction test. E: empty cup, S1: stranger 1. (*b*) Bar graph of social interaction times in WT (dark grey) and *Shank2^−/−^* (light grey) mice. *Shank2^−/−^* mice showed no preference between the stranger mouse and the empty cup in contrast to WT mice,which preferred the stranger mouse interaction. With high dietary zinc, social preference for interaction with the stranger mouse was restored in *Shank2^−/−^* mice. (*c*) Bar graph of social preference in WT (dark grey) and *Shank2^−/−^* (light grey) mice. Again, *Shank2^−/−^* mice showed significantly less preference for close interaction time with the stranger mouse compared with WT mice, and high dietary zinc reversed this phenotype such that social preference was now significantly higher than in *Shank2^−/−^* mice fed normal dietary zinc and no longer significantly different from WT. Each data point represents an individual animal (WT 30 ppm mice = 9, *Shank2^−/−^* 30 ppm mice = 9, WT 150 ppm mice = 11, *Shank2^−/−^* 150 ppm mice = 9). Blue data points are male mice and red data points are female mice. Data were analysed using (*b*) two-way analysis of variance (ANOVA) with Šídák’s *post hoc* test and (*c*) one-way ANOVA with Tukey’s *post hoc* test. ns = not significant. **p < 0.01, ***p < 0.001, ****p < 0.0001.

*Shank2*^−/−^ mice also showed a deficit in social interaction and social preference as measured by the time spent interacting between a stranger mouse and an empty cup (figure 2*a*). Specifically, *Shank2*^−/−^ mice showed no significant preference between the empty cup and the stranger mouse, in contrast to WT mice, which significantly preferred interacting with the stranger mouse (WT 30 ppm, stranger 1 = 123.1 ± 16.02 s, empty cup = 11.22 s, *p* < 0.0001; *Shank2*^−/−^ 30 ppm, stranger 1 = 26.89 ± 4.92 s, empty cup = 6.44 ± 1.725 s, *p* = 0.6610; figure 2*b*). With dietary zinc, however, *Shank2*^−/−^ mice showed a significant preference for the stranger mouse over the empty cup, as seen in WT mice (WT 150 ppm, stranger 1 = 124.5 ± 15.2, empty cup = 12.73 ± 4.427 s, *p* < 0.0001; *Shank2*^−/−^ 150 ppm, stranger 1 = 85.09 ± 23.93 s, empty cup = 14.27 ± 4.427 s, *p* = 0.0002, figure 2*b*). Calculation of the social preference index of close interaction time showed Shank2^−/−^ mice had a significantly lower social preference compared with WT mice (WT 30 ppm = 111.9 ± 16.63 s, *Shank2*^−/−^ 30 ppm = 20.44 ± 4.732 s; *p* = 0.0026; figure 2*c*). Again, with dietary zinc supplementation, the social preference index significantly increased in *Shank2*^−/−^ mice such that it was not significantly different from WT mice (WT 150 ppm = 111.8 ± 14.75 s; *Shank2*^−/−^ 150 ppm = 83.44 ± 23.63 s; *p* = 0.5956; figure 2*c*). As previously described [17], *Shank2*^−/−^ mice showed no significant phenotype for social novelty, i.e. interaction time between two stranger mice (data not shown; preference index for WT 30 ppm = 29.22 ± 11.01 s, *Shank2*^−/−^ 30 ppm = 10.44 ± 8.7 s, WT 150 ppm = 27.00 ± 9.07 s, *Shank2*^−/−^ 150 ppm 15.70 ± 10.61 s, *p* > 0.7 for multiple comparisons).

**Figure 3.**
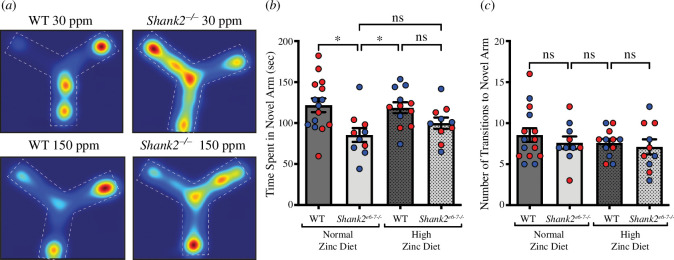
Y-maze testing shows a deficit in working memory in *Shank2^−/−^* mice that are not rescued by dietary zinc. (*a*) Heat maps representing example movements of animals during the Y-maze test. (*b*) Bar graph of time spent in the novel arm of the Y-maze in WT (dark grey) and *Shank2^−/−^* (light grey) mice. *Shank2^−/−^* mice spent significantly less time in the novel arm compared with WT mice. With high dietary zinc, *Shank2^−/−^* mice remained not significantly different from *Shank2^−/−^* mice fed a normal zinc diet. (*c*) No significant differences were observed across the WT and *Shank2^−/−^* mice with respect to the number of transitions into the novel arm of the Y-maze. Each data point represents an individual animal (WT 30 ppm mice = 15, *Shank2^−/−^* 30 ppm mice = 10, WT 150 ppm mice = 12, *Shank2^−/−^* 150 ppm mice = 10). Blue data points are male mice and red data points are female mice. All data were analysed using one-way ANOVA with Tukey’s *post hoc* test. ns = not significant. *p < 0.05.

We also performed the Y-maze test to examine the effects of dietary zinc supplementation on working memory in *Shank2^−/−^* mice (figure 3*a*–c). As previously shown, *Shank2^−/−^* mice show a deficit in working memory, spending significantly less time in the novel arm compared with WT mice (figure 3*b*; WT 30 ppm = 121.6 ± 8.3 s, *Shank2^−/−^* 30 ppm = 85.36 ± 8.521 s, *p* = 0.0102). Interestingly, this phenotype persisted with high dietary zinc, as *Shank2^−/−^* mice fed the high zinc diet did not return to control values and were not significantly different from *Shank2^−/−^* mice on a normal zinc diet (*Shank2^−/−^* 150 ppm = 99.89 ± 6.896 s; WT 30 ppm versus *Shank2^−/−^* 150 ppm *p* = 0.2132; *Shank2^−/−^* 30 ppm versus *Shank2^−/−^* 150 ppm *p* = 0.6266; figure 3*b*), showing that dietary zinc does not appear to influence this type of memory in these animals. No significant differences were observed in the number of transitions to the novel arm in either the WT or *Shank2^−/−^* mice group with either zinc diet (figure 3*c*).

**Figure 4.**
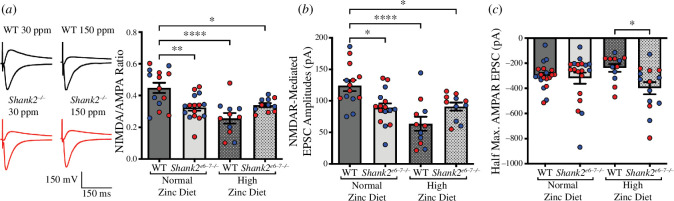
Electrophysiological analysis of NMDA/AMPA receptor-mediated currents in WT and *Shank2^−/−^* mice. (*a*) NMDA/AMPA EPSC ratios in WT and *Shank2^−/−^* mice fed normal (30 ppm) and high (150 ppm) dietary zinc. The NMDA/AMPA ratio was significantly decreased in *Shank2^−/−^* mice compared with WT controls, and high dietary zinc failed to return the ratio back to control levels. (*b*) NMDAR-mediated EPSC amplitudes at a stimulus intensity that stably evoked 200–350 pA AMPAR-mediated EPSC responses. NMDAR-mediated EPSC amplitudes were significantly reduced in *Shank2^−/−^* mice fed with a normal zinc diet (30 ppm) compared with WT controls and were not rescued by a high zinc diet (150 ppm). WT 30 ppm: 14 cells/6 mice, *Shank2^−/−^* 30 ppm: 16 cells/8 mice, WT 150 ppm: 11 cells/5 mice, *Shank2^−/−^* 150 ppm: 11 cells/6 mice (*a*) and (*b*). Data were analysed using one-way ANOVA with Tukey’s *post hoc* test for (*a*) and (*b*). (*c*) Half-maximal AMPAR EPSC amplitudes are not significantly altered in *Shank2^−/−^* mice with either high zinc diet. WT 30 ppm: 20 cells/9 mice, *Shank2^−/−^* 30 ppm: 19 cells/9 mice, WT 150 ppm: 12 cells/6 mice, and *Shank2^−/−^* 150 ppm: 14 cells/7 mice. Blue data points are male mice and red data points are female mice. Data were analysed using the non-parametric Kruskal–Wallis test. ns = not significant. *p < 0.05, **p < 0.01, ****p < 0.001.

We next performed hippocampal electrophysiological analysis in WT and *Shank2^−/−^* mice to examine whether glutamatergic synaptic function is also influenced by dietary zinc in these animals (figure 4). Measurements of the NMDA/AMPAR EPSC ratios revealed a significant decrease in *Shank2^−/−^* mice (WT 30 ppm versus *Shank2^−/−^* 30 ppm *p* = 0.0044; figure 4*a*). Dietary zinc was found to not reverse the significant effect on the NMDA/AMPA ratio in *Shank2^−/−^* mice fed the high zinc diet (WT 30 ppm versus *Shank2^−/−^* 150 ppm *p* = 0.0316; *Shank2^−/−^* 30 ppm versus *Shank2^−/−^* 150 ppm *p* = 0.9775). This phenotype was consistent with the measurement of NMDAR-mediated EPSC amplitudes alone (WT 30 ppm versus *Shank2^−/−^* 30 ppm *p* = 0.0136; WT 30 ppm versus *Shank2^−/−^* 150 ppm *p* = 0.0411; *Shank2^−/−^* 30 ppm versus *Shank2^−/−^* 150 ppm *p* = 0.999; figure 4*b*). Measurement of the half-maximal AMPAR-mediated EPSCs showed no significant difference between genotypes or zinc diet (WT 30 ppm = –299.4 ± 22.99 pA, *Shank2^−/−^* 30 ppm = –318.5 ± 44.41 pA, WT 150 ppm = –237.2 ± 30.80 pA, *Shank2^−/−^* 150 ppm = –398.9 ± 48.91 pA; *p* > 0.3 for multiple comparisons; WT 150 ppm versus *Shank2^−/−^* 150 ppm *p* = 0.0226; figure 4*c*), indicating that the failure of the high zinc diet to rescue the NMDA/AMPA ratio in *Shank2^−/−^* mice is caused by a lack of rescue of the decreased NMDAR-mediated response known to occur in *Shank2^−/−^* mice [8,17].

**Figure 5.**
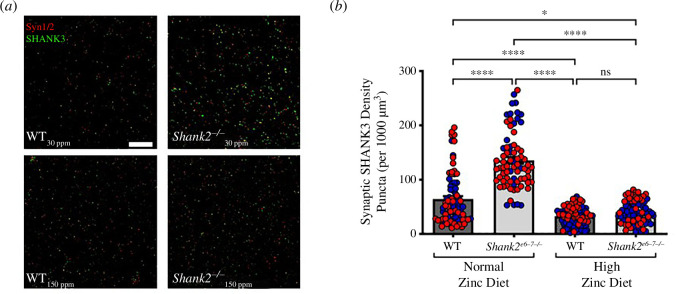
Immunohistochemical analysis of SHANK3 expression in WT and *Shank2^−/−^* mice. (*a*) Example confocal images of synapsin (red) and SHANK3 (green) hippocampal puncta expression in WT and *Shank2^−/−^* mice fed normal or high zinc diet. Scale bar = 10 µm. (*b*) Bar graph of the analysis of synaptic SHANK3 puncta density in WT (dark grey) and *Shank2^−/−^* (light grey) mice. *Shank2^−/−^* mice on a normal zinc diet showed significantly higher synaptic SHANK3 density compared with WT. Increasing dietary zinc resulted in a significant decrease in synaptic SHANK3 density in *Shank2^−/−^* mice. WT 30 ppm mice: *n* = 6; *Shank2^−/−^* 30 ppm mice: *n* = 6; WT 150 ppm mice: *n* = 6; and *Shank2^−/−^* 150 ppm mice: *n* = 6. Note that puncta were analysed from an average of three slices per animal with an average of four images taken per slice. Blue data points are male mice and red data points are female mice. Statistical differences were calculated with the D’Agostino and Pearson normality test for normally distributed data and with the Kruskal–Wallis (one-way ANOVA) test for multiple comparisons. ns = not significant, *p < 0.05, ****p < 0.0001.

We also performed immunohistochemical analysis to examine potential changes in SHANK3 expression at synapses (figure 5*a and b*) as our previous work had shown a zinc diet-dependent increase in SHANK3 recruitment to synapses that could be involved in dietary zinc rescue of synaptic function and ASD behaviours [13,14,16]. In contrast to *Tbr1^+/^*^−^ and *Shank3^−/−^* ASD mice, we observed that the synaptic SHANK3 density was significantly increased in *Shank2^−/−^* mice (WT 30 ppm ± 5.163 versus *Shank2^−/−^* 30 ppm 135.6 ± 6.020 puncta/1000 μm^3^; *p* < 0.0001). In response to high dietary zinc, and again in contrast to our previous work in *Shank3^−/−^* and *Tbr1^+/^*^−^ mice, the synaptic SHANK3 density was found to significantly decrease in *Shank2^−/−^* mice (*Shank2^−/−^* 150 ppm 40.36 ± 2.287 puncta/1000 μm^3^; *p* < 0.0001 in comparison to *Shank2^−/−^* 30 ppm mice; figure 5).

## 4. Discussion

Zinc is a major regulator of SHANK proteins at the synapse, and zinc deficiency has been shown to be prevalent in people affected with ASD [6,18–20]. This has led to the idea of a zinc link in ASD. We and others have shown that increasing zinc either through the diet or via zinc mobilization in ASD *Shank3^−/−^* and *Tbr1^+/−^* mouse models, or directly onto neuronal ± systems, can reverse or prevent ASD synaptic and behavioural deficits [6,13–17,19]. Here, we expanded this work to examine dietary zinc effects in *Shank2^−/−^* mice to determine whether similar efficacy is observed. Interestingly we have found differential effectiveness of dietary zinc in *Shank2^−/−^* mice. While we did observe a dietary zinc-induced reversal of hyperactivity and social preference, no effect was observed on working memory deficits. This form of memory is hippocampal-dependent, and this result correlated with a lack of rescue of the NMDA/AMPA ratio in *Shank2^−/−^* mice hippocampal neurons. Unlike in other dietary zinc studies, SHANK3 was not recruited to hippocampal synapses in response to increased zinc, but rather its expression was decreased, suggesting that zinc-dependent recruitment of SHANK3 is not involved in the rescue of ASD behaviours. Overall, our data show that *Shank2^−/−^* mice are differentially responsive to the effects of dietary zinc from the synaptic to the behavioural levels compared with *Shank3^−/−^* and *Tbr1^+/−^*.

Across the ASD mouse models examined to date, increasing zinc has been most effective on social interaction deficits across the *Shank2*^−/−^, *Shank3*^−/−^ and *Tbr1*^+/−^ mouse models [13,14,16,17]. This suggests that dietary zinc can influence the function of multiple brain regions including the prefrontal cortex, amygdala and hippocampus. Indeed zinc-induced synaptic effects and rescue of function have been observed in ASD models in the amygdala, hippocampus and striatum [13,14,16,17]. It is, therefore, of importance in future studies to expand the examination of dietary zinc effects in *Shank2*^−/−^ mice beyond the hippocampus to these additional brain regions. Zinc is a potent regulator of synaptic transmission, and previous studies have shown that zinc induces decreased synaptic expression and/or NMDAR channel conductance, as well as a change in NMDAR subunit expression [13,14,21–28]. Our previous studies examining the effects of dietary zinc have revealed that zinc-induced changes in synaptic function occur in both WT and ASD mice [13,14,16,17], and in the present study, we observed that dietary zinc decreased the NMDA/AMPA ratio in WT and *Shank2*^−/−^ mice. Previous work in male mice mobilizing zinc via clioquinol administration has shown restoration of NMDAR function in the hippocampus and rescue of LTP [17]. As we did not observe sex-specific differences in our experimental groups that included both male and female mice, the cause of the difference in NMDAR rescue observed in Lee *et al.* [17] and the lack of NMDA/AMPA ratio and working memory deficit rescue in the present study are likely a result of acute clioquinol application (2–3 h prior to measuring synaptic and behavioural effects) versus chronic (6 weeks) elevation of zinc, inducing distinct mechanistic changes that with dietary zinc do not appear to involve NMDAR and LTP rescue in the hippocampus. The chronic dietary zinc application also prevents targeting NMDAR hyperfunction observed at P14 in *Shank2*^−/−^ mice [29]. Given the developmental changes in NMDAR function from hyper- to hypofunction in *Shank2*^−/−^ mice [29], the acute versus chronic timing of changes in zinc and subsequent effects on synaptic and behavioural ASD deficits raise an additional factor to consider in targeting zinc for the rescue of ASD deficits as a potential therapeutic pathway.

The increase in SHANK3 at hippocampal synapses in *Shank2^−/−^* mice suggests a compensatory mechanism occurring at synapses with the lack of SHANK2. The subsequent decrease in SHANK3 with increased dietary zinc is unexpected given the evidence of zinc in recruiting SHANK3 to synapses. In *Shank3^−/−^* and *Tbr1^+/−^*, it was proposed that the recruitment of SHANK proteins with dietary zinc played a role in the rescue of synaptic and behavioural function[13,14,16]. The recruitment of SHANK3 is clearly not involved in the partial rescue of function in *Shank2^−/−^* mice as SHANK3 was elevated in the mutant mice with normal dietary zinc alongside the presence of significant ASD deficits. As SHANK3 is known to drive the recruitment of post-synaptic proteins including AMPA and NMDA receptors to synapses [30], this may contribute to the lack of NMDAR rescue in hippocampal neurons. Therefore, we hypothesize that other zinc-responsive proteins are involved in the dietary zinc effects in *Shank2^−/−^* mice, and these may include zinc-responsive pre-synaptic proteins given the ability of SHANKs to regulate pre-synaptic protein recruitment and pre-synaptic function [30].

The heterogeneity of dietary zinc effects across ASD mouse models examined to date suggests that differential efficacy occurs with ASD genetic variants. As ASD is defined as a broad spectrum of deficits in social and communication behaviours that can be caused by a large range of genetic variants, it is potentially not surprising that each ASD variant will have a different responsivity profile. Currently, the effects observed from the synaptic to the behavioural level are limited to animal ASD models, so there is a limited ability to extrapolate to dietary zinc efficacy in human ASD genetic variants. Our data however do support the current idea that development of therapeutic targets, especially in severe forms of ASD, may require patient-specific causative genetic information to effectively target relevant pathways.

## Data Availability

The data can be accessed at [31].
